# Contextualized Clinical Practice Guidelines for Poststroke Motor Rehabilitation in India: Framework and Endorsement for Implementation

**DOI:** 10.1159/ced/accag013

**Published:** 2026-08-28

**Authors:** Dorcas B.C. Gandhi, Rinita Mascarenhas, Elizabeth Lynch, Coralie English, Julie Bernhardt, Jeyaraj D. Pandian, John M. Solomon

**Affiliations:** a Department of Physiotherapy, Manipal College of Health Professions, Manipal Academy of Higher Education, Manipal, India; b Department of Neurology, Christian Medical College and Hospital, Ludhiana, India; c Caring Futures Institute, College of Health and Enablement, Flinders University, Adelaide, SA, Australia; d School of Health Sciences, University of Newcastle, Newcastle, NSW, Australia; e Centre of Research Excellence to Accelerate Stroke Trial Innovation and Translation, University of Sydney, Sydney, NSW, Australia; f Florey Institute of Neuroscience and Mental Health, Heidelberg, VIC, Australia

**Keywords:** Stroke rehabilitation, Clinical practice guidelines, Contextualization, Motor rehabilitation, India

## Abstract

**Introduction:**

Existing Indian stroke rehabilitation clinical practice guidelines (CPGs) focus predominantly on physiotherapy and provide limited guidance on health system integration, care pathways, and implementation. This study aimed to develop an evidence-based, contextually relevant CPG for motor rehabilitation across the stroke continuum in India, identify priority recommendations, and assess implementation feasibility.

**Methods:**

A three-phase mixed-methods study was conducted across tertiary, secondary, and community-based rehabilitation settings in India. A scoping review, focus group discussions, and systematic synthesis of existing guidelines were undertaken. Participants included rehabilitation professionals with ≥10 years of experience, persons with lived experience of stroke, and key stakeholders. Recommendations were classified according to evidence strength and implementation feasibility.

**Results:**

Rehabilitation pathways were fragmented, with limited post-discharge access and no standardized referral systems. Sixty clinical questions were refined into nine PICO questions and two practice points. Eighty-four recommendations were synthesized from high-quality international guidelines meeting AGREE-II/AGREE-REX thresholds of ≥60%. Using a modified Alper tool, 50 recommendations supported interventions, 13 advised against routine use, and the remainder had limited or unclear evidence. Using the Adopt-Contextualize-Adapt framework, 39 recommendations were suitable for adoption, 32 required contextualization, and 13 required adaptation.

**Conclusion:**

A stakeholder-informed, contextually adapted stroke rehabilitation CPG was developed for India, addressing key evidence and implementation gaps and providing a scalable framework to improve rehabilitation quality, continuity, and accessibility.

## Introduction

Clinical practice guidelines (CPGs) are tools that translate evidence into actionable recommendations for healthcare providers [1, 2]. However, CPGs from many low- and middle-income countries (LMICs) lack methodological rigor, multidisciplinary input, and contextual relevance [3]. Practitioners from LMICs report that many CPGs are difficult to apply in routine practice and within local healthcare constraints [4].

In India, a lower middle-income country, where the crude prevalence of stroke ranges from 26 to 757/100,000 people annually, the government launched the National Stroke Care Guidelines through the Indian Council of Medical Research and proposed a National Stroke Action Plan integrating stroke care into national health programs [5–9]. Despite this, the 2019 and 2022 Indian stroke rehabilitation CPGs focus largely on physiotherapy and do not address India’s health system, patient care pathways, or implementation challenges [7, 10]. These CPG development teams included doctors of neurology, community medicine, physical medicine and rehabilitation, research scientists, technical/partnership/financial consultants, and administrators from the Indian Ministry of Health and Family Welfare. The process of guideline development was not described in the document, and a lack of stakeholder engagement was apparent, with no involvement of therapy teams, patients, caregivers, or administrators, increasing the risk of limited practical relevance. The absence of evidence grading, implementation, and dissemination strategies further undermines uptake [3]. These weaknesses are reflected in low Appraisal of Guidelines REsearch and Evaluation (AGREE)-II (8.45) and AGREE-Recommendations Excellence (REX) (1.23) scores reported by the Global Consortium for Stroke Rehabilitation [3].

Effective implementation of evidence requires consideration of local resources, infrastructure, provider skills, patient pathways, and health system variability [8–11]. The importance of describing the usual patient pathway after stroke was first demonstrated in the Philippines [12] and subsequently used in contextualizing stroke rehabilitation guidelines in South Africa [13]. Patient pathways are a way to describe the typical sequence of care experienced by patients in a defined context, reflecting real-world service availability, referral patterns, and resource constraints. They can inform the development of feasible implementation strategies for evidence-based rehabilitation [12, 13].

To develop stroke rehabilitation guidelines that can be feasibly implemented in India’s complex health system, similar methods were used in this study. As an initial step, this study focuses on motor rehabilitation, with the following aims:

1. Describe the current usual-care patient pathway for poststroke motor rehabilitation in India
2. Develop a comprehensive, evidence-based, India-contextualized CPG for motor rehabilitation across acute, subacute, and chronic stroke phases.

## Methods

This study is reported based on the Standards for Reporting Qualitative Research (SRQR) checklist [14].

### Aim 1: Describe the Usual-Care Patient Pathway for Poststroke Motor Rehabilitation in India

We used a mixed-methods approach to integrate existing evidence and relevant stakeholder insights.

- A scoping review was conducted to explore the determinants affecting access to and utilization of poststroke motor rehabilitation in LMICs. This review has been published, including reporting of the determinants affecting stroke rehabilitation in India [10]. The review identified three major categories of determinants: (1) contextual factors, (2) personal factors, and (3) resource-related factors. These findings informed the stakeholder discussions, patient pathway mapping, and contextualization process undertaken in the present study.
- Data collection: To integrate stakeholder insights, three online focus groups were conducted. Participants were stroke rehabilitation professionals with a minimum of 10 years of clinical experience in India, two persons with lived experience of stroke who had undergone rehabilitation in India within the past 5 years, and one health policy advisor. Participants were recruited through professional networks and recognized national bodies, ensuring representation from different regions (urban, semi-urban, rural) and healthcare sectors (public and private) (Table 1)

**Table 1.** Focus group participant characteristics

Participant ID	Qualification/stroke survivor	Professional role	Years of experience	Health sector	Area of expertise
P1	Neurology	Senior consultant and professor	27	Tertiary care, public	Stroke, acute care, and implementation
P2	Physical medicine and rehabilitation	HoD and consultant	25	Tertiary care, public	Stroke rehabilitation, spasticity management, etc.
P3	Physiotherapy	Senior clinical PT	15	Tertiary care, public	Acute care stroke rehabilitation and telerehabilitation
P4	Physiotherapy	Associate professor and administrator	22	Tertiary care, private	Stroke rehabilitation and clinical/translational research
P5	Physiotherapy	Home-based PT	11	Rural home-based care	Stroke rehabilitation
P6	Physiotherapy	Home and clinic/OPD-based PT	12	Rural and semi-urban home-based care	Stroke rehabilitation and vocational training
P7	Occupational therapy	Clinician and assoc. professor	15	Secondary care, public	Chronic poststroke rehabilitation
P8	Rehabilitation nurse	Ward in-charge	10	Secondary, private	Neurological disorders
P9	Stroke survivor	–	Ongoing rehabilitation	At a local clinic/OPD	–
P10	Stroke survivor	–	Ongoing rehabilitation	At a tertiary care hospital	–
P11	Public health expert	Policy advisor	10	Public sector, India	Implementation sciences and finance in health/primary care

HoD, head of the department; PT, physiotherapist; OPD, outpatient department.

The focus groups were facilitated by the first author using a semi-structured discussion guide (online suppl. material 1; for all online suppl. material, see https://doi.org/10.1159/ced/accag013). The focus group discussion (FGD) included primarily open-ended questions designed to elicit explanations of participants’ experiences with stroke motor rehabilitation, their patient care pathway, and questions associated with clinical practices. All participants provided informed consent prior to participating. All sessions were recorded with consent, and verbatim transcriptions were made.

- Data analysis: Thematic saturation was considered to have been achieved when findings from the focus groups showed substantial convergence with the determinants and themes identified in the scoping review, with limited emergence of novel themes beyond those already identified in the literature. Using thematic analysis, focus group data were inductively coded and iteratively organized into larger themes and subsequently subthemes. The emergent themes aimed to capture the differences in stroke rehabilitation pathways across the Indian healthcare system. Data from our scoping review [10] were triangulated with the emergent themes to enhance trustworthiness. When findings from the two sources diverged, neither was considered the definitive source of truth. Instead, both sources were treated equally, and discrepancies were systematically examined to identify any contextual differences, gaps in current literature, or new perspectives from the focus group participants. Divergences are reported in online supplementary material 2. Convergences across the 2 sources strengthened the validity of the interpretations. Independent coding by multiple team members and cross-verification of interpretations further reinforced methodological rigor and improved trustworthiness.
- Qualitative analysis also included extracts from the FGDs and the scoping review being coded against Theoretical Domain Framework-version 2 (TDF2) domains.

### Aim 2: Develop a Comprehensive, Evidence-Based, India-Contextualized CPG for Motor Rehabilitation

Step A: Generation and refinement of clinical questions (Fig. 1)

- Data collection: During the FGDs addressing aim 1, participants identified 60 clinical questions on poststroke motor rehabilitation relevant to the Indian context. These questions represented areas of uncertain evidence and/or evidence that clinicians find difficult to implement. Clinical questions were systematically mapped to poststroke timelines (acute, early subacute, late subacute, chronic) [15, 16] (online suppl. material 3).
- Refinement process: Step 1 – An initial review removed duplication and overlap, yielding 35 discrete clinical questions; step 2 – these were refined into standardized Population, Intervention, Comparison, Outcome (PICO) format through three iterative rounds of expert discussion by all authors based on selection criteria listed below and finalized 17 PICOs for further development (online suppl. material 3). Selection decisions were reached through consensus among the investigators rather than through a formal quantitative scoring system, consistent with the exploratory and contextual nature of the study:
  - Inclusion criteria:
    1. PICOs relevant to poststroke motor rehabilitation.
    2. PICOs with recommendations that could feasibly be contextually adapted and applied within the Indian healthcare setting or similar LMICs.
  - Exclusion criteria: PICOs with recommendations that could not be implemented or measured within a 6-month timeframe in the context of phase 3 of the study.

**Fig. 1.**
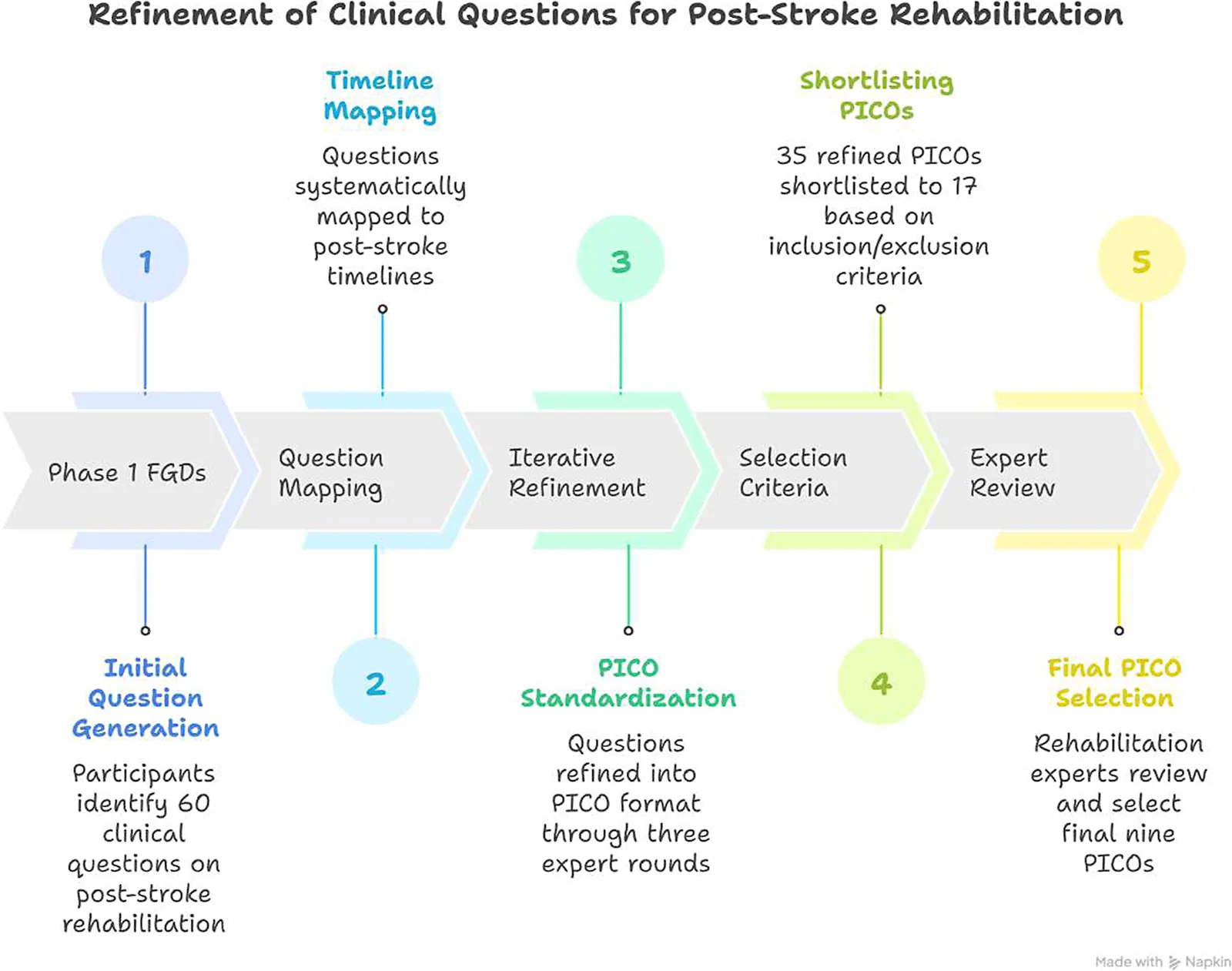
PICO development and refinement.

• Step 3: Finally, with input from two rehabilitation experts (J.S. and J.B.), the PICOs were reviewed to determine whether they should be combined or kept distinct. This process resulted in the final selection of nine PICOs (Table 2) for which solutions/recommendations were to be developed.

- To ensure rigor and validity, the iterative process involved multiple expert inputs (investigator triangulation) in refining PICOs with multiple rounds of review (methodological triangulation) for finalizing PICOs. The mapping of questions to the stroke pathway also served as a form of analytical triangulation, reinforcing that the clinical queries were grounded in real-world practice.

**Table 2.** List of 9 finalized PICO questions

Serial no.	9 PICO questions	Domains addressed	Stage of stroke rehab pathway (Fig. 4)
1	What are the most effective strategies to rehabilitate upper limb function in the in-hospital phase poststroke	Sensory, motor, functional abilities, ADLs, and QoL	In-hospital
2	What are the most effective ways in preventing a subluxation?	Secondary complications	All
3	What techniques can most effectively improve walking?	Sensory, motor, functional abilities, ADLs, and QoL	All
4	What strategies can increase intensity and duration of active use of the affected upper limb in activities	Therapy dosage	All
5	What interventions maximize QoL in those with severe strokes and who are bed-bound?	Sensory, motor, functional abilities, ADLs, and QoL	Home or community based
6	How to reduce secondary complications for patients with severe strokes? (severe stroke defined according to NIHSS or FMA score, where these are unavailable, mRS score, where none of these are available, define severe strokes as those unable to sit or stand without assistance, severe hemiparesis and reduced level of consciousness)	Sensory, motor, functional abilities, ADLs, and QoL	All
7	What strategies can improve general mobility (bed mobility defined as all mobility on a bed and out-of-bed mobility as bed to chair, bed to stool and back, etc.) in a stroke survivor?	Secondary complications	All
8	What strategies can improve independence for ADLs	Sensory, motor, functional abilities, ADLs, and QoL	Home or community based
9	What are the effective management strategies to improve function and/or limit pain in those with central pain or CRPS poststroke	Mode of therapy delivery	Home or community based
​	Two practice points	​	​
1	What basic medical criteria (e.g., hemodynamic stability, resolution of intracranial pressure) should determine readiness for motor rehabilitation initiation?	–	In-hospital
2	What caregiver-led rehabilitation programs improve function in stroke survivors?	–	Home or community based

QoL, quality of life; NIHSS, National Institutes of Health Stroke Scale; FMA, Fugl-Meyer Assessment; CRPS, complex regional pain syndrome; ADL, activity of daily living.

### Focus Group Discussions

Step B: Contextualization of evidence-based recommendations for poststroke motor rehabilitation

- Development of tier 1 evidence: A comprehensive database of evidence-based recommendations relevant to poststroke motor rehabilitation was developed by the lead author by synthesizing high-quality CPGs. This work was conducted in coordination with the World Stroke Organization’s (WSO) initiative on developing global quality indicators for stroke rehabilitation. Only CPGs that scored ≥60% on both the AGREE-II and AGREE-REX appraisal tools were included, ensuring methodological rigor and relevance in addition to two other documents (D.G.). The following sources contributed to the database:
  1. Australian New Zealand Living Guidelines for Stroke Management (rehabilitation) [16].
  2. European Stroke Organization (ESO) Guidelines for Motor Rehabilitation [17].
  3. Canadian Best Practice Recommendations for Stroke Rehabilitation [18].
  4. WSO-Lancet Neurology Commission’s Pragmatic Solutions for Stroke Rehabilitation [19].
  5. South African Contextualized Stroke Rehabilitation Guideline [13, 20].

For each of the nine PICOs identified in phase 2, corresponding recommendations were extracted, including the strength and quality of the supporting evidence.

- Data analysis and interpretation: An Indian adaptation of a decision-making tool (Fig. 2) was developed based on the modified Alper guide [20, 21] to determine the composite strength of each available recommendation across CPGs [13].This decision-making tool (modified Alper decision-making tool-Indian version) takes into consideration the number, consistency, and quality of the CPGs. In addition, we considered the local context, including risks/benefits of the recommendation. Using this approach, a finalized set of contextualized recommendations was generated, each assigned a composite strength-of-evidence rating. The strengths of body of evidence were classified as consistently weak or consistently strong based on the level of evidence, while consistency was based on the number of CPGs favoring the recommendation that were either recommending for or against an intervention. The strength of each recommendation was shown with tick √ marks. A working example is elaborated in online supplementary material 4.
- Framework for contextualization and endorsement: To explore the feasibility of implementation of each recommendation within the Indian context, the Adopt, Contextualize, Adapt (ACA) framework was applied [13] by three independent raters. This structured framework allowed each recommendation to be classified into three possible implementation pathways (ACA) [13].
  a. Adopt (implemented as-is)
    - Within 6 months A1
    - With long-term organizational or policy changes A2
  b. Contextualize (implemented after addressing local barriers)
    - Within 6 months B1
    - With long-term support plans B2
  c. Adapt (requires additional local evidence or expert consensus)
    - If secondary research of existing evidence is feasible C1
    - If secondary research is not feasible, develop consensus-based guidance C2.

**Fig. 2.**
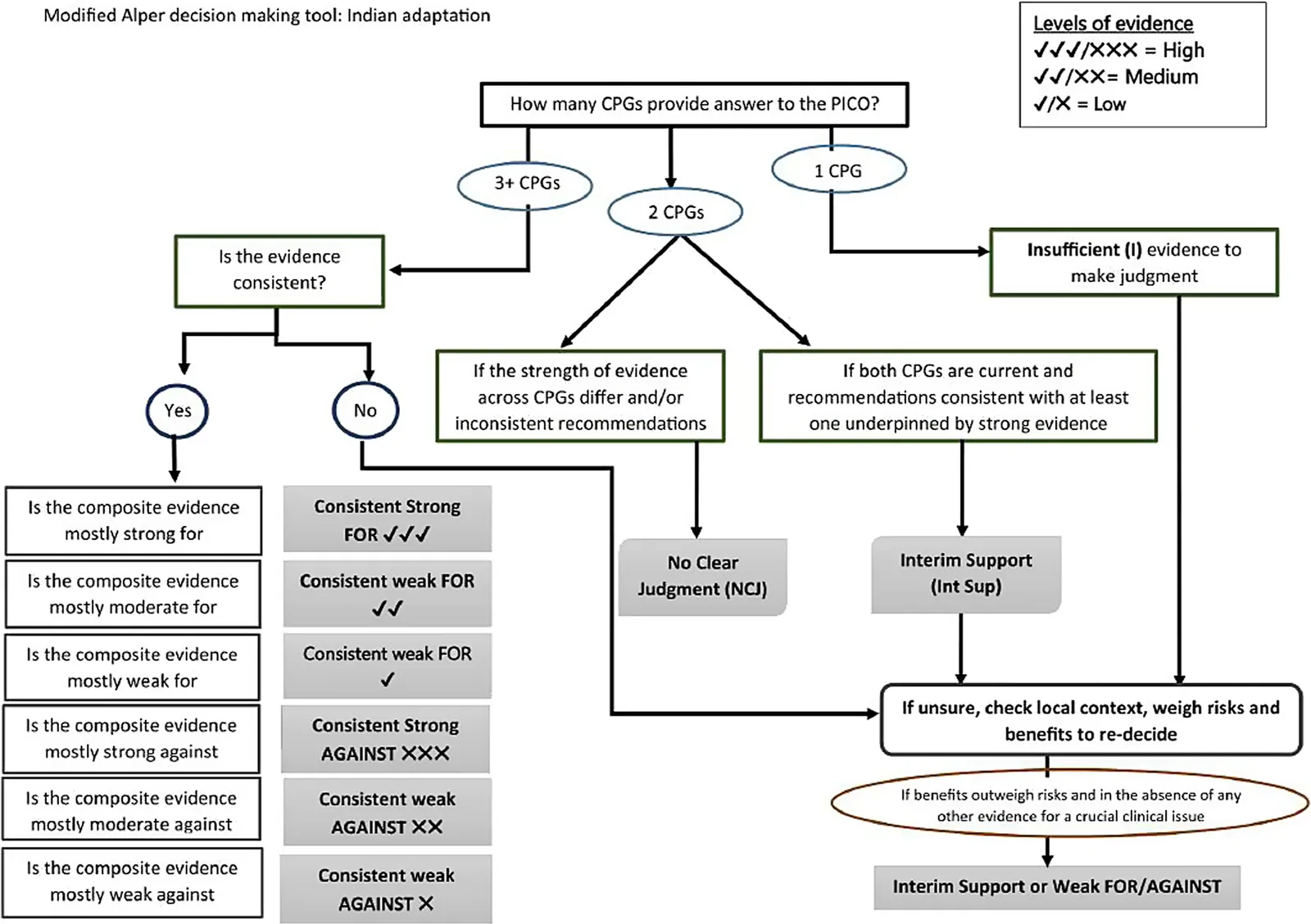
Modified Alper decision-making tool: Indian adaptation.

Three independent stroke rehabilitation experts from India (D.G., R.M., and M.N.) assessed each recommendation using the ACA framework. A majority vote among the experts determined the final endorsement level for each recommendation. As the aim of this process was pragmatic contextualization rather than formal psychometric validation, inter-rater agreement statistics were not calculated. Where disagreements occurred (*n* = 3), and all three experts assigned different endorsement levels, the final endorsement level was determined based on the strength of the overall body of evidence. Recommendations supported by strong evidence, either in favor of or against the intervention, were assigned the highest endorsement level proposed by the experts to facilitate early implementation. We have illustrated the contextual CPG development process in Figure 3.

**Fig. 3.**
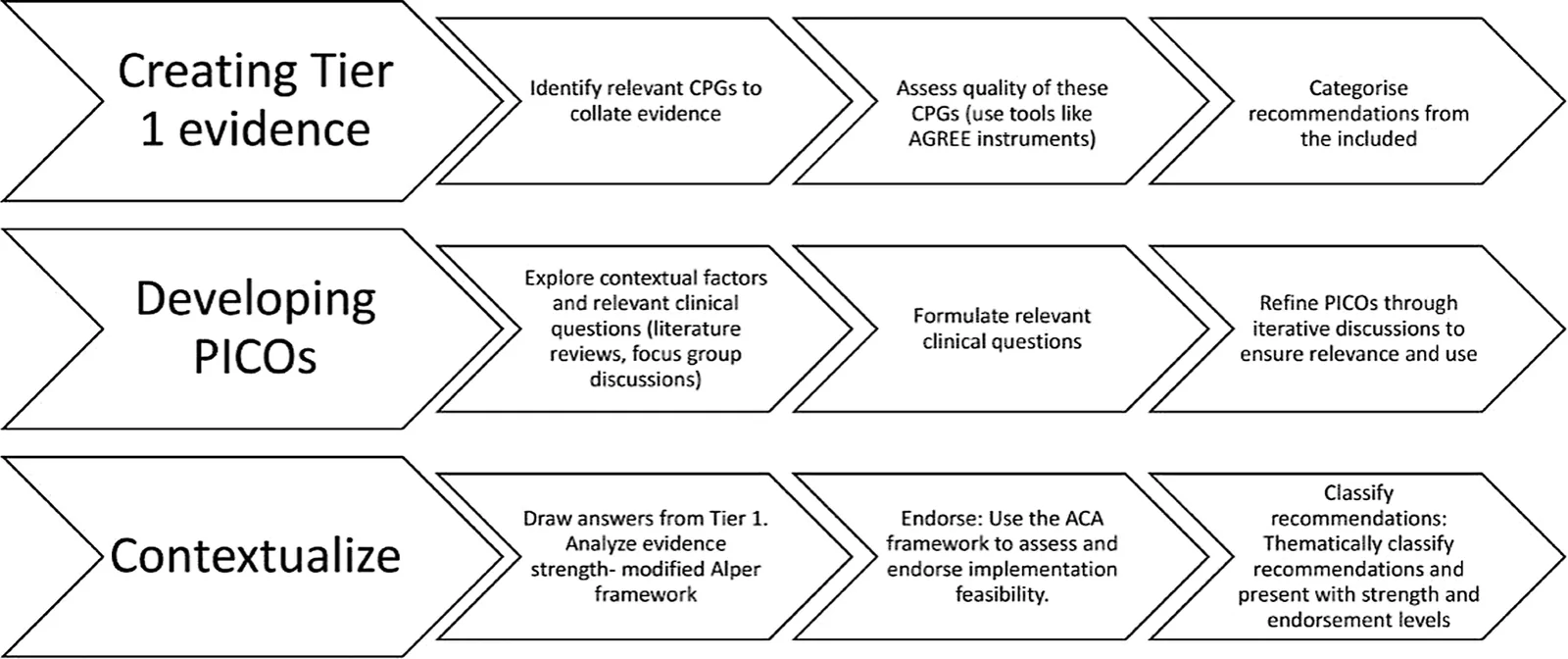
Steps involved in the development process of contextual CPGs.

## Results

### Aim 1: Stroke Rehabilitation Pathway

Findings from the FGDs revealed that there was no consistent rehabilitation referral system for survivors of stroke in India. The various pathways to rehabilitation identified are illustrated in Figure 4. The pathway presented was generated based on the inputs of the FGDs and was reviewed by the expert participants (online suppl. material 2).

**Fig. 4.**
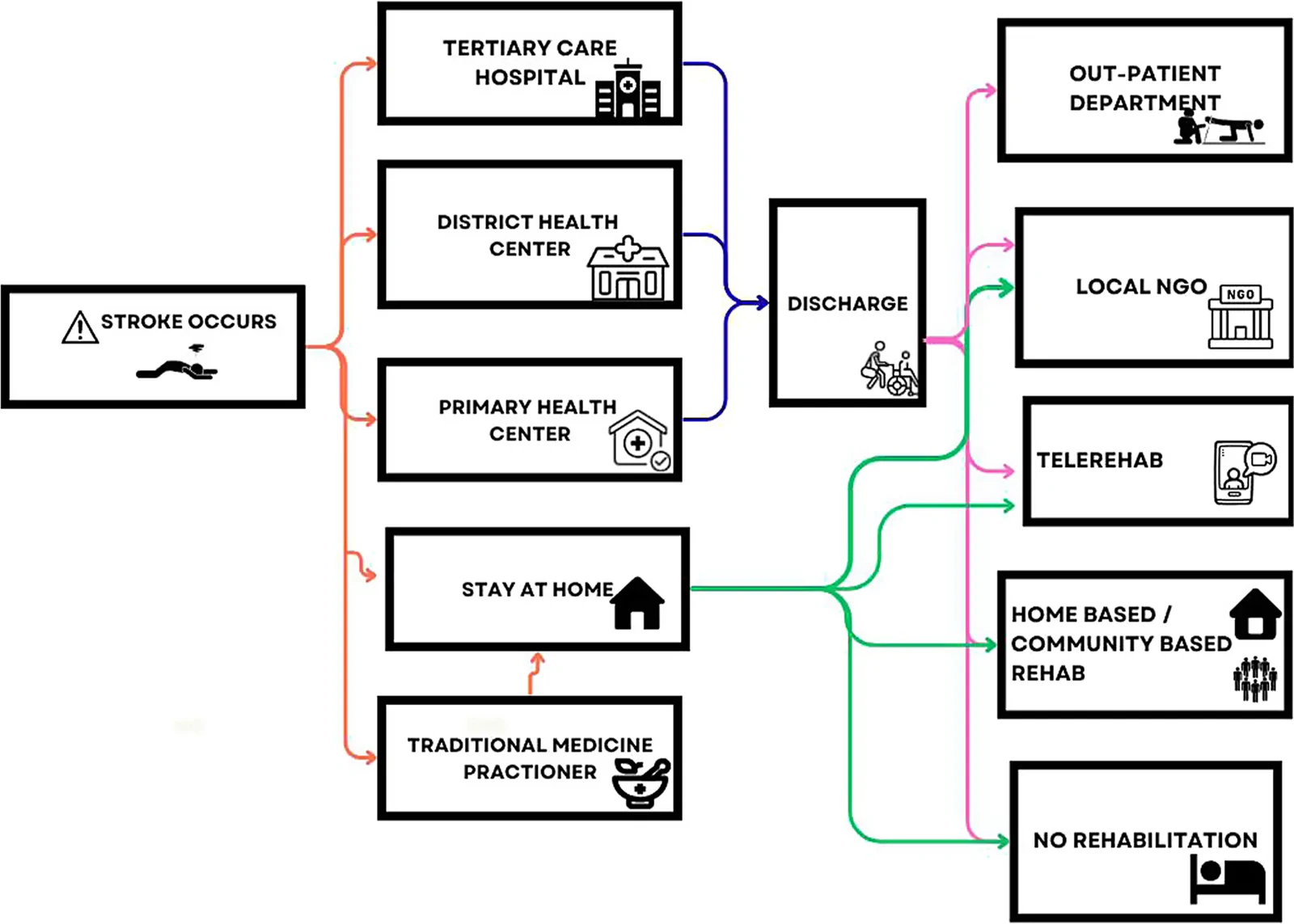
Stroke rehabilitation pathways in India.

Focus group participants (Table 1) stated that following discharge from acute care, most patients either transition to home-based rehabilitation or discontinue rehabilitation altogether. Within hospitals, the content and dose of stroke rehabilitation differ based on the level of healthcare. In the Indian healthcare system, primary care provides first-contact basic services at the community level, secondary care offers specialist and inpatient services through referrals, and tertiary care delivers advanced, super-specialty treatment with high-end infrastructure and expertise. We found that in tertiary care hospitals, particularly those affiliated with teaching institutions, physiotherapy services are typically available but there is often a lack of services from other healthcare disciplines. The quality content and intensity of rehabilitation are difficult to quantify due to limited data. At secondary-level and primary-level facilities, rehabilitation is further constrained, focused primarily on acute medical care, and minimal therapy is provided, contingent on various determinants [10]. The contextualized recommendations developed in this study are mapped to the different patient pathways described.

We also mapped 31 coded extracts from the FGDs to the TDF2 domains, mostly identified barriers rather than enablers. The strongest TDF domains are Environmental Context and Resources and Knowledge, showing that resource constraints and unclear guidance are the main issues. Key barriers include staff shortages, infrastructure gaps, cost, transport, limited equipment, and poor access to specialist rehabilitation. The coding suggests that implementation needs practical protocols, training, caregiver/CHW support, workflow prompts, and audit feedback.

### Aim 2: Step A – List of PICOs

Nine PICO questions were finalized in alignment with the study aims and predefined eligibility criteria (Table 2). In addition, two questions with potential to influence routine clinical practice were identified and were therefore reported as practice points. No existing recommendations addressing practice point 1 were identified within tier 1 evidence sources, including high-quality international CPGs.

### Aim 2: Step B – Contextualization

a. Evidence mapping and recommendation distribution

A total of 84 recommendations (online suppl. material 5) were extracted from tier 1 evidence and mapped across nine PICOs and two practice points. Most recommendations addressed prevention and management of secondary complications (*n* = 26), followed by sensory-motor impairments (*n* = 14) related to the upper limb and mobility. Recommendations related to discharge planning and community reintegration accounted for 11 statements. Cognition and communication were addressed through 14 recommendations, including speech and language interventions (*n* = 9) and cognitive rehabilitation focusing on attention, memory, and apraxia (*n* = 5). Activity limitation and quality of life were targeted by 13 recommendations. Fewer recommendations addressed therapy dosage and goal setting (*n* = 3), early rehabilitation and mobilization (*n* = 2), and mode of therapy delivery, including telehealth (*n* = 1).

b. Strength of evidence and endorsement for implementation

In 84 recommendations, we found 50 were in favor of intervention, 13 were against routine use, 11 had insufficient evidence, 8 were unclear, and 1 needed interim support. One recommendation was classified as a practice point. With respect to endorsement levels, 13 recommendations required adaptation (C1 = 8; C2 = 5). C1 recommendations included 4 weak for, 3 insufficient, and 1 no clear judgement variant. C2 recommendations included 2 weak for, and 3 no clear judgement recommendations.

Thirty-two recommendations required contextualization (B1 = 15; B2 = 17). Among B1 recommendations, 3 were strong for, 7 weak t for, 3 insufficient, 1 no clear judgement, and 1 interim support. Among B2 recommendations, 3 were consistent strong for, 10 weak for, 3 insufficient, and 1 no clear judgement. Twenty-one recommendations were suitable for direct adoption.

Within A1 recommendations (*n* = 25), 6 were t strong for, 5 consistent strong against, 7 weak against, and 4 weak for. In addition, A1 contained 1 no clear judgement and 2 insufficient recommendations. Within A2 recommendations (*n* = 14), 5 were strong for and 7 weak for. One recommendation was classified as no clear judgement, and one as a practice point. Table 3 lists all the strong recommendations with endorsement levels of A1 or A2 with the rest of the 84 recommendations listed in online supplementary material 5.

**Table 3.** All strong recommendations (for and against) with A1/A2 endorsement levels

Stage of the stroke rehab pathway	Categories	Final list of recommendations	Final evidence strength	Final endorsement level
All	Upper and lower extremity: impairment	For stroke survivors with reduced strength in their arms or legs, progressive resistance training should be provided to improve strength	Consistent strong FOR ✔✔✔	A1
In-hospital and community	Goal setting	Health professionals should initiate the process of setting goals and involve stroke survivors and their families and carers throughout the process. Goals for recovery should be client-centered, clearly communicated and documented so that both the stroke survivor (and their families/carers) and other members of the rehabilitation team are aware of goals set. Goals should be set in collaboration with the stroke survivor and their family/carer (unless they choose not to participate) and should be well defined, specific, and challenging. They should be reviewed and updated regularly	Consistent strong FOR ✔✔✔	A2
All	Upper extremity: impairment	Hand and wrist orthoses (splints) should not be used as part of routine practice as they have no effect on function, pain, or range of movement	Strong consistent AGAINST ✘✘✘	A1
All	Spasticity and contractures	For stroke survivors at risk of developing contracture who are receiving comprehensive, active therapy, the routine use of splints or stretch of the arm or leg muscles is not recommended	Strong consistent AGAINST ✘✘✘	A1
All	Task specific training – standing	For stroke survivors who have difficulty in standing up from a chair, practice of standing up should be undertaken	Consistent strong FOR ✔✔	A1
All	Task specific training – standing	For stroke survivors who have difficulty with standing, activities that challenge balance should be provided	Consistent strong FOR ✔✔	A1
All	Activity limitation	Administration of amphetamines to improve ADL is not recommended	Strong AGAINST ✘	A1
In-hospital and community	DVT	Antithrombotic stockings are not recommended for the prevention of DVT or PE poststroke	Consistent strong AGAINST ✘✘✘	A1
In-hospital	Nutrition and hydration	All patients with stroke should have their hydration status assessed, monitored, and managed throughout their hospital admission. Where fluid support is required, crystalloid solution should be used in preference to colloid solutions as the first option to treat or prevent dehydration	Consistent strong FOR ✔	A2
In-hospital	Nutrition and hydration	All patients with stroke should be screened for malnutrition at admission and on an ongoing basis (at least weekly) while in hospital	Consistent strong FOR ✔✔	A1
In-hospital	Nutrition and hydration	For patients with stroke whose nutrition status is poor or deteriorating, nutrition supplementation should be offered	Consistent strong FOR ✔✔	A1
In-hospital	Early mobilization	For stroke patients, starting intensive out-of-bed activities within 24 h of stroke onset is not recommended	Consistent strong AGAINST ✘	A1
In-hospital	Early mobilization	All stroke patients should commence mobilization (out-of-bed activity) within 48 h of stroke onset unless otherwise contraindicated (e.g., receiving end-of-life care)	Consistent strong FOR ✔	A1
All	Task specific training – sitting	For stroke survivors who have difficulty sitting, practicing reaching beyond arm’s length while sitting with supervision/assistance should be undertaken	Consistent strong FOR ✔	A2
In-hospital	Speech and language	For stroke survivors with aphasia, early aphasia therapy, starting within the first 4 weeks poststroke should be provided to maximize language recovery	Consistent strong FOR ✔	A2
All	Speech and language	Communication partner training should be provided to health professionals or volunteers who interact with people with aphasia after stroke	Consistent strong FOR ✔✔	A2

ADL, activity of daily living; ✓, low level of evidence; ✓✓, medium level of evidence; ✓✓✓, high level of evidence.

## Discussion

In this study, we adopted a systematic and structured process to develop a contextualized CPG for motor rehabilitation poststroke for India. We adapted and applied the modified Alper guide, by adding a step of evaluating the risk-to-benefit ratio of those recommendations supported by limited or insufficient evidence. This allowed for an inclusive approach, enabling consideration of recommendations supported by even a single CPG, where appropriate, especially in the local context and in the absence of locally relevant evidence.

The methodological approach used in this study aligns with, and extends, prior efforts to contextualize rehabilitation guidelines in LMICs, notably in the Philippines and South Africa [12, 13]. Similar to these initiatives, our process combined evidence synthesis with stakeholder engagement and explicitly described the usual patient pathway as a foundation for contextualization. This study advances previous models in three key ways: (1) the modified Alper decision-making tool incorporated the consistency and quality of evidence in addition to consideration of local risks and benefits. This allowed recommendations supported by limited evidence to be retained where clinically justified and contextually appropriate. (2) The structured application of the ACA framework by independent raters provided transparent endorsement categories, which may facilitate implementation planning and may improve usability for clinicians and policymakers. (3) Mapping recommendations to India’s healthcare levels and to real-world patient pathways may strengthen alignment between evidence, service delivery capacity, and policy planning. Collectively, these refinements make the applicability of the methodology replicable and scalable within India and potentially other LMICs. Additionally, the study provides a guide to the number and profile of stakeholders in the contextualization process (Table 1) and the criteria for selecting tier 1 evidence (refer to step B of aim 2 of this manuscript) even in contexts/countries where high-quality evidence may not exist. This further supports the need for such contextualization initiatives rather than generating new evidence in every context/country.

Figure 2 provides a replicable methodological model that may be relevant to other LMICs facing similar challenges to those observed in India, such as limited skilled workforce, the absence of uniform rehabilitation standards, and poor access to rehabilitation services [2, 21, 22]. The development and use of such contextualized CPGs as described in this paper could be a potential solution to address these gaps and improve rehabilitation service delivery. The practical utility of this methodology is already reflected in its application to the development of a poststroke physiotherapy guide by the Indian Association of Physiotherapists. Additionally, the patient pathway developed through this study may support the interpretation and implementation of recommendations in relation to the real-world rehabilitation journey of people with stroke.

This step-wise and adaptive approach may support ease of use by clinicians, policymakers, funders, nonmedical professionals, and organizations, and can assist in prioritizing evidence for implementation with available resources and local systems. Contextual recommendations have the potential to inform insurance coverage and reimbursement frameworks under the “Pradhan Mantri Jan Arogya Yojana” (PMJAY) [22, 23] and similar schemes, particularly for outpatient and home-based rehabilitation services currently not reimbursed in India. Similarly, contextualized CPG could also be used as a performance metric within the country in national registries and hospital accreditation standards to improve accountability and thus implementation success. However, these potential applications require further validation through implementation and health systems research. This model is grounded in stakeholder engagement, and context specificity improves adoption nationally and by other LMICs that wish to improve stroke rehabilitation systems and to fall in line with the WHO 2030 agenda [24].

## Limitations

This study has several limitations. Although stakeholder engagement was purposive and multidisciplinary, the number of participants, particularly persons with lived experience of stroke and policymakers, was limited, which may not fully capture the heterogeneity of patient journeys and administrative realities across India. Additionally, recruitment was through professional networks, which may have introduced selection bias. Given the substantial interstate, tier-based, and public-private variability in India’s health system, local deviations from the usual-care pathway are inevitable. Third, the quality, intensity, and dose of rehabilitation services could not be quantified due to the absence of routine national rehabilitation data, limiting the ability to benchmark pathways against measurable service standards. Local primary evidence remains sparse; thus, the recommendations, although contextualized, are from high-income countries. This raises a need for future research in this field. This is also reflected in the absence of primary research on readiness for initiation of motor rehabilitation, and caregiver-led rehabilitation programs improve function in stroke survivors that were raised a practice-related questions during the FGDs. The next phase of this work is planned to focus on implementation and evaluation of prioritized recommendations in India at 2 hospitals in India. Implementation research methods, including hybrid effectiveness-implementation designs, will be employed to evaluate impact on patient outcomes and service delivery processes. Development of implementation tools such as healthcare-level-specific care bundles, training modules, and audit indicators will be included to operationalize the recommendations.

## Future Scope

Targeted systematic reviews and primary studies are needed to address identified evidence gaps especially for the practice points, and particularly around caregiver-led rehabilitation, task-shifting, management of shoulder subluxation, criteria to initiate early mobilization. Finally, integration of these contextualized CPGs into national programs, insurance reimbursement frameworks, accreditation standards, and professional training curricula are required to achieve sustained scale-up. Policy implications are substantial: the healthcare-level-mapped pathway provides a blueprint for rational workforce planning, task-sharing, and resource allocation across primary, secondary, and tertiary levels; supports integration of rehabilitation indicators into national stroke registries and accreditation standards.

## Conclusion

This study presents a transparent, stakeholder-informed, and methodologically rigorous approach to developing contextualized CPGs for poststroke motor rehabilitation in India. Grounded in real-world patient pathways and mapped to the country’s healthcare level, integrating high-quality international evidence with locally derived priorities and endorsement levels, we provide structured contextualization that is replicable across LMICs and offers an evidence-informed basis for expanding reimbursement under publicly funded insurance schemes to include outpatient and home-based rehabilitation. In a setting where access remains fragmented and rehabilitation intensity is inconsistent, contextualized CPGs represent a potential systems-level intervention. However, the impact of these contextualized recommendations on patient outcomes, service delivery, equity, and long-term quality of life requires further evaluation through future implementation and effectiveness research. Further embedding these recommendations within national programs and implementation research platforms is essential to translate evidence into measurable gains of life for stroke survivors in India and comparable LMICs.

## Acknowledgments

The authors would like to thank all participants who contributed to this study.

## Statement of Ethics

This study was conducted in accordance with the ethical principles of the Declaration of Helsinki. Ethical approval was obtained from the Institutional Research Committee of Kasturba Medical College and Kasturba Hospital, Manipal Academy of Higher Education (MAHE), Manipal, India (Approval No. IEC1:410/2023). Written informed consent was obtained from all participants before enrolment in the study.

## Conflict of Interest Statement

The authors have no conflicts of interest to declare.

## Funding Sources

This study was supported by the Society of Indian Physiotherapists. The funding body had no role in the design of the study; data collection, analysis, or interpretation; manuscript preparation; or the decision to submit the manuscript for publication.

## Author Contributions

Dorcas B.C. Gandhi: conceptualization, methodology, supervision, formal analysis, investigation, writing, review, and editing. Rinita Mascarenhas: data curation, formal analysis, investigation, and writing – original draft. Elizabeth Lynch and Coralie English: methodology, writing, review, and editing. Julie Bernhardt: supervision, review, and editing. Jeyaraj D. Pandian: supervision and review. John M. Solomon: conceptualization, methodology, supervision, writing, review, and editing. All authors have read and approved the final manuscript and met the ICMJE criteria for authorship.

## Data Availability

All data generated or analyzed during this study are included in this article and its online supplementary. Further inquiries can be directed to the corresponding author.
